# Pareidolias are a function of visuoperceptual impairment

**DOI:** 10.1371/journal.pone.0293942

**Published:** 2023-11-06

**Authors:** Emily McCann, Soohyun Lee, Felicia Coleman, John D. O’Sullivan, Peter J. Nestor

**Affiliations:** 1 Queensland Brain Institute, The University of Queensland, St Lucia, Queensland, Australia; 2 UQ Centre for Clinical Research, Faculty of Medicine, The University of Queensland, Herston, Queensland, Australia; 3 Department of Neurology, Royal Brisbane & Women’s Hospital, Herston, Queensland, Australia; 4 Mater Neurosciences Centre, Mater Hospital, South Brisbane, Queensland, Australia; Clinical Investigation Center, TUNISIA

## Abstract

Pareidolias, or the misperception of ambiguous stimuli as meaningful objects, are complex visual illusions thought to be phenomenologically similar to Visual Hallucination (VH). VH are a major predictor of dementia in Parkinson’s Disease (PD) and are included as a core clinical feature in Dementia with Lewy Bodies (DLB). A newly developed Noise Pareidolia Test (NPT) was proposed as a possible surrogate marker for VH in DLB patients as increased pareidolic responses correlated with informant-corroborated accounts of VH. This association could, however, be mediated by visuoperceptual impairment. To understand the drivers of performance on the NPT, we contrasted performances in patient groups that varied both in terms of visuoperceptual ability and rates of VH. *N* = 43 patients were studied of whom *n* = 13 had DLB or PD with Dementia (PDD); *n* = 13 had PD; *n* = 12 had typical, memory-onset Alzheimer’s Disease (tAD); and *n* = 5 had Posterior Cortical Atrophy (PCA) due to Alzheimer’s disease. All patient groups reported pareidolias. Within the Lewy body disorders (PD, DLB, PDD), there was no significant difference in pareidolic response rates between hallucinating and non-hallucinating patients. Visuoperceptual deficits and pareidolic responses were most frequent in the PCA group—none of whom reported VH. Regression analyses in the entire patient cohort indicated that pareidolias were strongly predicted by visuoperceptual impairment but not by the presence of VH. These findings suggest that pareidolias reflect the underlying visuoperceptual impairment of Lewy body disorders, rather than being a direct marker for VH.

## Introduction

Visual hallucination (VH) is a core clinical feature of Dementia with Lewy Bodies (DLB) [1] and a predictor in Parkinson’s Disease (PD) of conversion to dementia (PDD) [2]. PD, PDD and DLB can be grouped together collectively as Lewy body disorders (LBD). VH in LBD are associated with cholinergic degeneration, particularly in posterior isocortical regions [3, 4], and can be improved with cholinesterase inhibitors [5, 6]. Currently, VH are assessed through clinical interview with the patient and, ideally, corroborated by an informant. The assessment of VH, therefore, can be somewhat subjective; patients may be unreliable or not forthcoming while informants rely, in large part, on what patients report to them.

The Noise Pareidolia Test (NPT) has been proposed as a potential surrogate marker of VH in patients with neurodegenerative diseases [7]. Pareidolias, the misperception of ambiguous information as meaningful objects, are thought to be phenomenologically similar to VH [8, 9]. There have been a range of cognitive paradigms proposed to elicit pareidolias [10, 11]. This study focused on the NPT because it has had the most clinical uptake, particularly in LBDs as a surrogate marker for VH. In the NPT, patients are asked to report the presence or absence of a face embedded in visual noise—a face is actually present in 8/40 exemplars with the remainder comprised of noise only [7]. A pareidolia is recorded when the patient misidentifies noise as a face. Previous research has shown that pareidolic responses correlated with VH presence in both PD [12] and DLB patients [7, 9], though the association was less pronounced in those thought to have mild cognitive impairment due to Lewy body disease [13]. In DLB, both pareidolic responses and VH decreased after starting a cholinesterase inhibitor [9]. Interestingly, pareidolic responses have also correlated with clinical tests of visuoperception [9, 12]. Given that across the spectrum of LBD (PD, PDD and DLB): (i) VH are associated with dementia or the imminent emergence of dementia and (ii) that visuoperceptual dysfunction is a key feature of LBD-associated dementia, it remains unclear whether the pareidolic response in the NPT is truly a measure of the propensity to hallucinate or, rather, a sign of visuoperceptual impairment.

In the current study, we aimed to determine if pareidolias are specifically a marker of VH, or if they are driven by visuoperceptual impairment independent of VH. A challenge to answering this question is that, in LBD, VH and visuoperceptual dysfunction are likely to be strongly associated with each other making it difficult to unpick the behavioural underpinning of pareidolias. Therefore, to help dissociate the influence of VH from visuoperceptual dysfunction in the generation of pareidolias, we also studied patients with Alzheimer’s disease (AD) because in this disease the emergence of visuoperceptual deficits is typical, whereas VH is relatively uncommon. Furthermore, we included both the typical, memory-onset (tAD), and Posterior Cortical Atrophy (PCA), phenotypes. PCA is known to have extreme, higher order visual dysfunction [14]—the inclusion of both AD phenotypes offered a wide range of visuoperceptual performance, ideal for regression analyses.

We hypothesized that pareidolias may actually be a sign of visuoperceptual impairment and, consequently, that their association with VH is an epiphenomenon. We also predicted that LBD patients experiencing VH would have worse visuoperceptual ability compared to those that have not yet developed VH.

## Materials and methods

### Participants

43 patients participated in the study between January 2022 and April 2023, including PD without dementia (*n* = 13); PD with dementia (PDD) or DLB (*n* = 13); typical Alzheimer’s Disease (tAD; *n* = 12); and Posterior Cortical Atrophy (PCA; *n* = 5). All patient diagnoses were confirmed using diagnostic criteria (DLB: [1]; PCA: [15]; PD: [16]; PDD: [17]; tAD: [18]) by experienced neurologists (JDOS, PJN). Those in the tAD and PCA groups had additional biomarker confirmation of Alzheimer pathology by cerebrospinal fluid examination. As PDD (*n* = 5) and DLB (*n* = 8) have similar cognitive and neuropsychiatric profiles [19], these patients were collapsed into a single group. All patients were tested while on their usual medication and those with an LBD were always tested in the “on” state. Patients were classified as hallucinators when they reported a history of well-formed, complex VH, corroborated by their caregivers. If patients were taking a cholinesterase inhibitor that suppressed their previously experienced VH, they were still classified as a hallucinator. All participants had their visual acuity measured with the Snellen chart. N = 32 healthy volunteers (see Table 1 for demographics) who were screened to exclude cognitive impairment, neurological and psychiatric illness, and had normal or corrected-to-normal vision, were also administered the NPT. Level of education was converted to the International Standard Classification of Education (ISCED) [20]. Participant demographics are presented in Table 1.

**Table 1:** Demographics for participants in the pareidolia study.

	HC	tAD	PCA	PD	DLB-PDD	Omnibus Significance (*p*)
n	32	12	5	13	13	
Age	67.3±7.1	66.8±6.5	67.8±4.5	67.5±8.2	75.7±5.4	0.005^§^^a^^b^
Gender (M | F)	17 | 15	6 | 6	4 | 1	8 | 5	10 | 3	0.474
Education (ISCED)	5.2±2.1	3.7±1.6	3.0±1.6	4.7±1.7	3.2±2.4	0.136
Visual Acuity (Median [Range])	6/6 [6/5–6/12]	6/6 [6/5–6/18]	6/24 [6/9–6/24]	6/7.5 [6/6–6/18]	6/9 [6/6–6/12]	0.078
Symptom Duration		3.2±1.0	6.4±5.3	4.3±2.6	6.5±3.9	0.096
H&Y (/5)				1.5±0.5	1.7±0.4	0.245
ChI (%)	0	42	40	8	92	< 0.001^b^
Antidepressants (%)*		33	20	8	46	0.172
LEDD				646.2±417.5	296.2±139.3	0.06
History of VH (%)	0	0	0	0	77	< 0.001^a^^b^^c^

*Abbreviations*. ChI = Cholinesterase Inhibitor; H & Y = Modified Hoehn and Yahr scale; ISCED = International standard classification of education; LEDD = Levodopa Equivalent Daily Dose [21]; VH = Visual Hallucinations.

* included: Selective Serotonin Reuptake Inhibitors, Serotonin and Norepinephrine Reuptake Inhibitors, and mirtazapine

^§^ ANOVA

^a^ tAD < DLB-PDD

^b^ PD < DLB-PDD

^c^ PCA < DLB-PDD

Patients with an LBD completed the Modified Hoehn and Yahr scale (H&Y) [22]. The DLB-PDD group was significantly older than the PD and tAD groups; were prescribed more cholinesterase inhibitors compared to the PD group; and had a greater prevalence of hallucinations compared to tAD, PCA, and PD groups.

### General neuropsychology

The Addenbrooke’s Cognitive Examination III (ACE-III) [23] and the Montreal Cognitive Assessment (MoCA) [24] were used as global cognitive scales. Participants also performed specific neuropsychological tests of visuoconstruction: Rey-Osterrieth Complex Figure (ROCF) copy [25, 26]; visuoperception using the Cube Analysis (CA), Incomplete Letters (IL), and Progressive Silhouettes (PS) subtests from the Visual Object and Space Perception battery (VOSP) [27]; memory using Rey Auditory Verbal Learning Test (RAVLT) [28] and recall of the ROCF; naming using the naming subtest of the Sydney Language Battery (SYDBAT) [29]; attention and working memory were assessed using the Forward and Backward Digit Span Test (Digit Span) [30] and the Digit Symbol Substitution test (DSST) [31]. The results are summarised in Table 2.

**Table 2:** General neuropsychology.

	HC	tAD	PCA	PD	DLB-PDD	Omnibus Significance (*p*)
ACE-III						
*Total (/100)*	94.0±3.5	72.4±14.8	45.4±17.3	90.8±8.5	75.3±11.8	< 0.001 ^a^^b^^c^
*Attention (/18)*	17.0±1.0	12.9±3.2	10.4±3.6	16.8±1.5	13.9±3.1	0.001 ^a^^b^^c^
*Fluency (/14)*	12.4±1.4	8.7±3.3	5.0±4.7	11.8±2.4	7.5±3.8	0.004 ^b^^c^
*Language (/26)*	24.9±1.4	23.1±2.5	15.0±7.8	25.3±1.0	22.5±4.5	0.001 ^a^^b^^c^
*Memory (/26)*	24.4±1.8	14.7±6.3	12.2±6.2	21.6±4.9	19.0±4.6	0.005 ^a^^b^
*Visuospatial (/16)*	15.3±1.0	13.1±2.7	2.8±1.1	15.2±1.0	12.5±2.4	< 0.001 ^b^^c^^d^
MoCA (/30)	26.2±1.1	18.1±4.2	12.8±4.9	25.3±3.0	19.5±2.9	< 0.001^§^^a^^b^^c^^d^^e^
Digit Span (/28)	16.6±4.7	12.4±5.4	10.2±3.5	14.8±3.0	12.4±5.2	0.232^§^
Naming (/30)	27.0±1.7	22.1±3.4	16.8±7.5	26.5±3.4	22.3±3.8	0.002 ^a^^b^^c^
DSST	53.1±12.4	27.6±18.0	0.0±0.0	39.8±10.8	15.3±11.2	< 0.001^§^^b^^c^^d^
Rey Auditory Verbal Learning Test (RAVLT)						
*Immediate Recall (/15)*	13.0±2.8	5.8±2.7	5.8±1.0	10.3±3.4	6.8±3.0	0.002 ^§^^a^^b^^c^
*Delayed Recall (/15)*	11.3±4.5	1.2±1.0	1.8±2.4	7.3±4.4	4.5±2.6	<0.001^a^^b^^f^
Rey-Osterrieth Complex Figure						
*Copy (/36)*	33.2±1.7	22.8±10.3	3.0±0.6	28.5±8.9	17.6±10.3	< 0.001 ^b^^c^
*Recall (/36)*	15.1±4.8	2.2±2.2	0.8±0.9	14.4±8.8	5.5±3.6	< 0.001 ^a^^b^
Visual Object and Space Perception Battery						
*Cube Analysis (/10)*	9.2±1.0	7.3±2.2	0.2±0.4	9.4±1.0	5.6±2.5	< 0.001 ^b^^c^^d^
*Incomplete Letters (/20)*	19.7±0.5	18.8±1.5	1.8±3.0	19.5±0.9	14.5±5.0	< 0.001 ^b^^c^^d^^g^
*Progressive Silhouettes (/20)*	11.0±2.0	10.8±2.3	16.6±4.6	8.9±2.8	12.2±2.5	< 0.001^§^^h^^i^^j^^k^

*Abbreviations*. ACE-III = Addenbrooke’s Cognitive Examination-III; DSST = Digit Symbol Substitution Test; MoCA = Montreal Cognitive Assessment; *derived from in-house database of *n* = 115 healthy controls (age 65.7±8.1).

^§^ANOVA

^a^ tAD < PD

^b^ PCA < PD

^c^ PD > DLB-PDD

^d^ tAD > PCA

^e^ PCA < DLB-PDD

^f^ tAD < DLB-PDD

^g^ tAD > DLB-PDD

^h^ tAD < PCA

^i^ PCA > PD

^j^ PCA > DLB-PDD

^k^ PD < DLB-PDD

#### Noise Pareidolia Test [9]

The NPT was completed by all participants. The test included 40 visual noise trials, of which eight contained a black-and-white human face. Three practice trials were completed to familiarise participants with the task—feedback was provided on practice trials only. For each stimulus, participants indicated the presence and location of a face. Responses were classified as correct when participants indicated the absence of a face on noise-only trials, or indicated the true location of a face on face-present trials. When participants incorrectly reported the absence of a face on a face-present trial, the response was classified as a miss. Conversely, indicating a face present on a noise-only trial was classified as a pareidolia. If multiple faces were incorrectly reported on a noise-only trial, multiple pareidolias were recorded. Indicating the incorrect location of a face on a face-present trial was also classed as a pareidolia.

### Statistical analysis

Statistical analyses were performed using RStudio version 4.2.0. Shapiro-Wilk tests were performed to determine data normality. Group-wise comparisons were performed using parametric one-way Analysis of Variance (ANOVA) with Tukey post-hoc tests, or non-parametric Kruskal-Wallis tests with Dunn post-hoc tests (omnibus statistics in the Tables are Kruskal-Wallis tests except where specified). A visuoperception composite score was created by averaging the three VOSP subtests (the PS was reverse scored because, unlike the other two VOSP subtests, higher scores indicate worse performance) to explore the relationship between pareidolic responses and visuoperceptual ability using Spearman’s rank correlation coefficient. Finally, a linear regression with one categorical variable (positive or negative history for VH) and one continuous variable (the visuoperception composite score) was run to explore predictors of the pareidolic scores.

## Results

### Noise Pareidolia Test

Performance of all groups on the NPT is shown in Table 3.

**Table 3:** Performance on the Noise Pareidolia Test.

	HC	tAD	PCA	PD	DLB-PDD	Omnibus Significance (*p*)
Correct Responses (/40)	38.4±2.4	35.0±8.2	16.6±12.1	35.8±6.3	32.3±8.7	0.005^a^^b^
Misses (/8)	0.2±0.4	0.3±0.5	4.0±1.6	0.2±0.6	0.9±0.9	< 0.001^c^^d^^e^
Pareidolias	1.4±2.4	5.0±8.2	26.6±23.4	3.9±6.3	7.1±9.4	0.013^c^^d^

^a^ tAD > PCA

^b^ PCA < PD

^c^ tAD < PCA

^d^ PCA > PD

^e^ PCA > DLB-PDD

**Fig 1:**
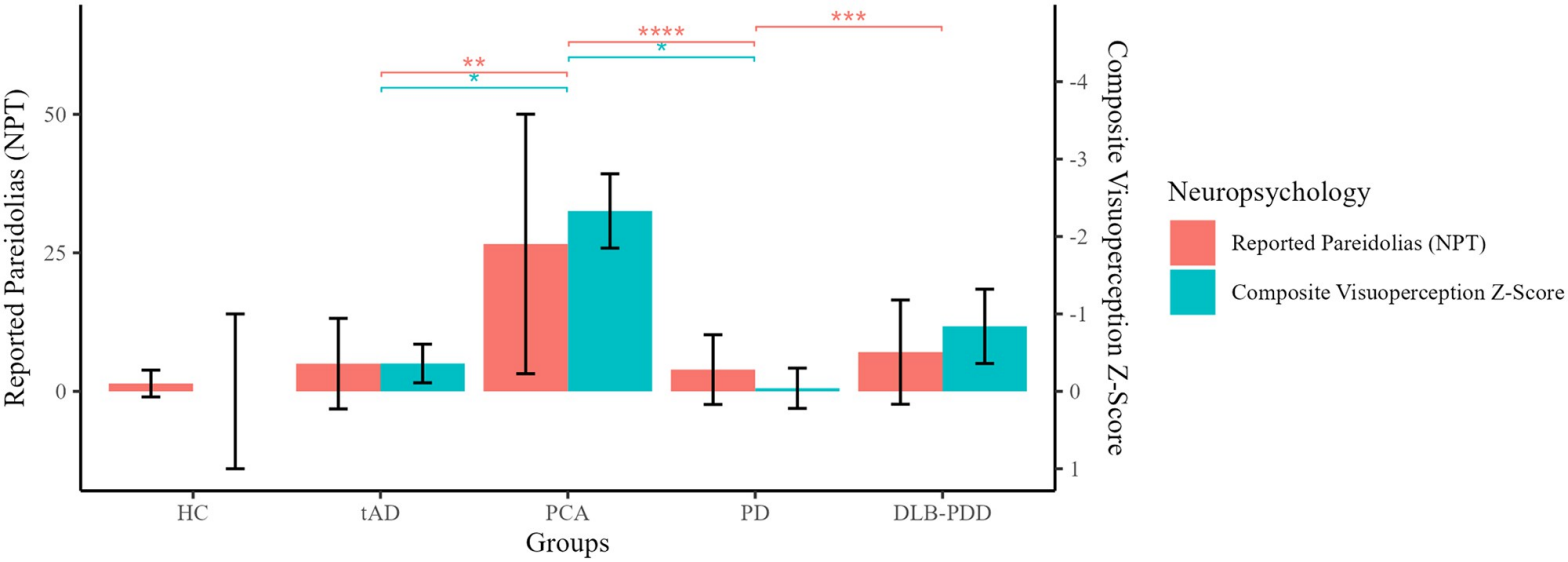
The composite visuoperception score, and pareidolic responses across patient groups. *Note*. The composite visuoperception scores are inverted to positive for ease of comparison to the pareidolia score thus a greater visuoperception z-score corresponds to greater impairment. * p < 0.05; ** p < 0.01; *** p < 0.001; **** p < 0.0001.

There were significant differences on the NPT (Kruskal-Wallis tests) across patient groups for correct responses (H(3) = 12.7, *p* = 0.005); misses (H(3) = 20.5, *p* = 0.001); and pareidolic responses (H(3) = 10.7, *p* = 0.013). Holm-corrected, Dunn’s post hoc tests revealed that the PCA group gave significantly less correct responses compared to the PD group (*p* = 0.002) and tAD groups (*p* = 0.002). The PCA group missed more faces compared to tAD (*p* = 0.003), and the PD group (*p* < 0.001). The PCA group reported the highest number of pareidolic responses reaching statistical significance compared to the PD group (*p* = 0.035) and tAD group (*p* = 0.049; Fig 1).

### Visuoperception

There were significant differences across groups (Kruskal-Wallis) on the composite visuoperception measure, H(3) = 29.1, *p* < 0.001. After adjusting for multiple comparisons, Dunn’s tests revealed that the PD group had better performance than the PCA (*p* < 0.001) and DLB-PDD groups (*p* < 0.001) on this measure (Fig 1). The tAD group had better visuoperception scores compared to the PCA group, *p* = 0.004).

### Comparison of hallucinators and non-hallucinators in LBD (PD, PDD and DLB combined)

Across all patients with LBD, Spearman’s correlation, corrected for multiple comparisons, showed a significant negative relationship between history of VH, and disease severity as measured using the ACE-III, (ρ = -0.65, *p* = 0.001). There was a non-significant trend for a relationship between VH and pareidolic responses (ρ = 0.30, *p* = 0.14).

**Fig 2:**
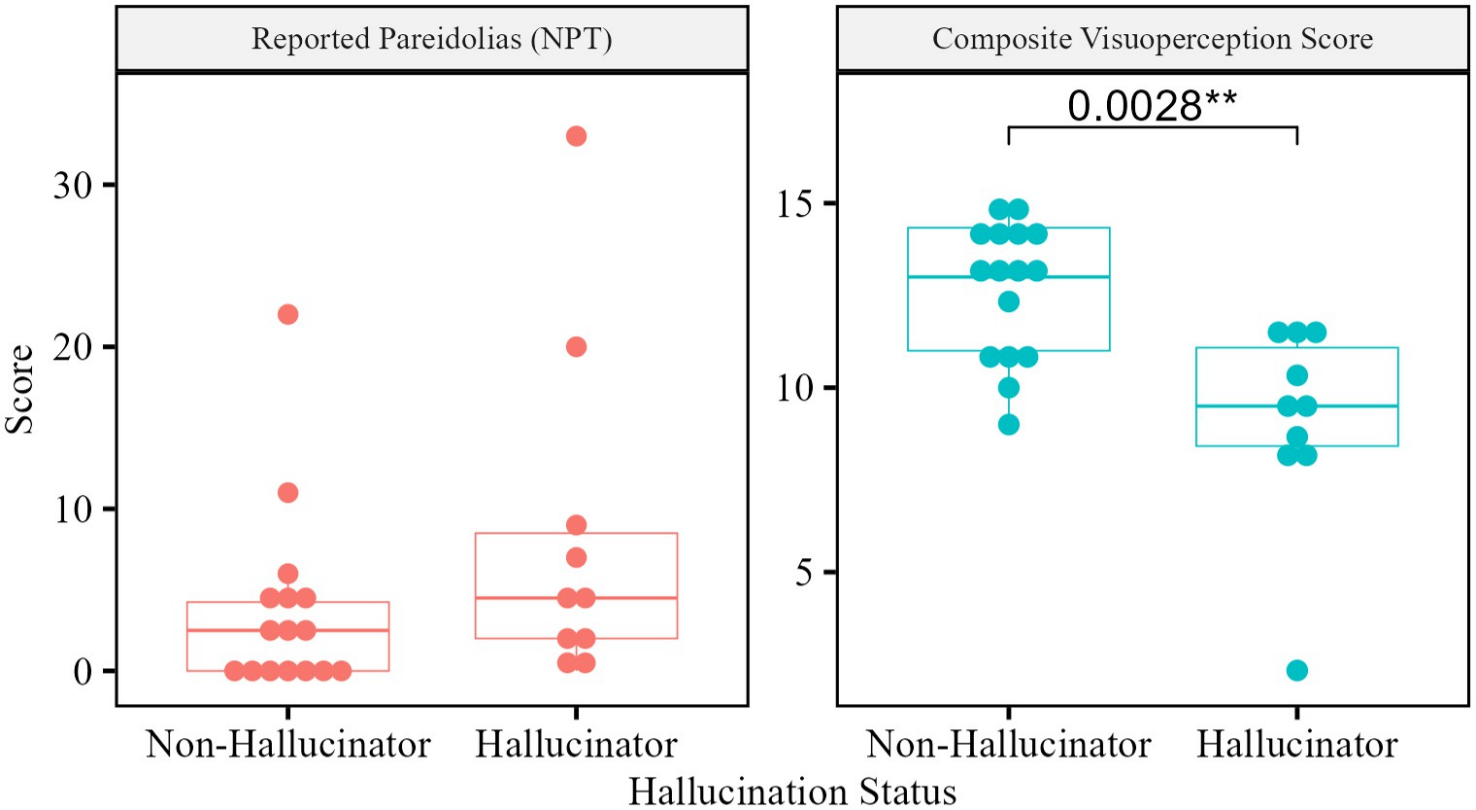
Composite visuoperception and pareidolia scores in LBD patients with and without a history of VH.

A Wilcoxon signed-rank test determined a significant difference in visuoperception score when LBD patients had a history of VH (*n* = 10, median = 9.50), compared to those without (*n* = 16, median = 13.0), *z* = - 3.19, *p* = 0.001 (Fig 2). There was a non-significant difference in pareidolic responses between hallucinating (median = 4.5) and non-hallucinating (median = 2.5) LBD patients, *z* = - 1.47, *p* = 0.14.

### Correlations of pareidolias in all patient groups

**Fig 3:**
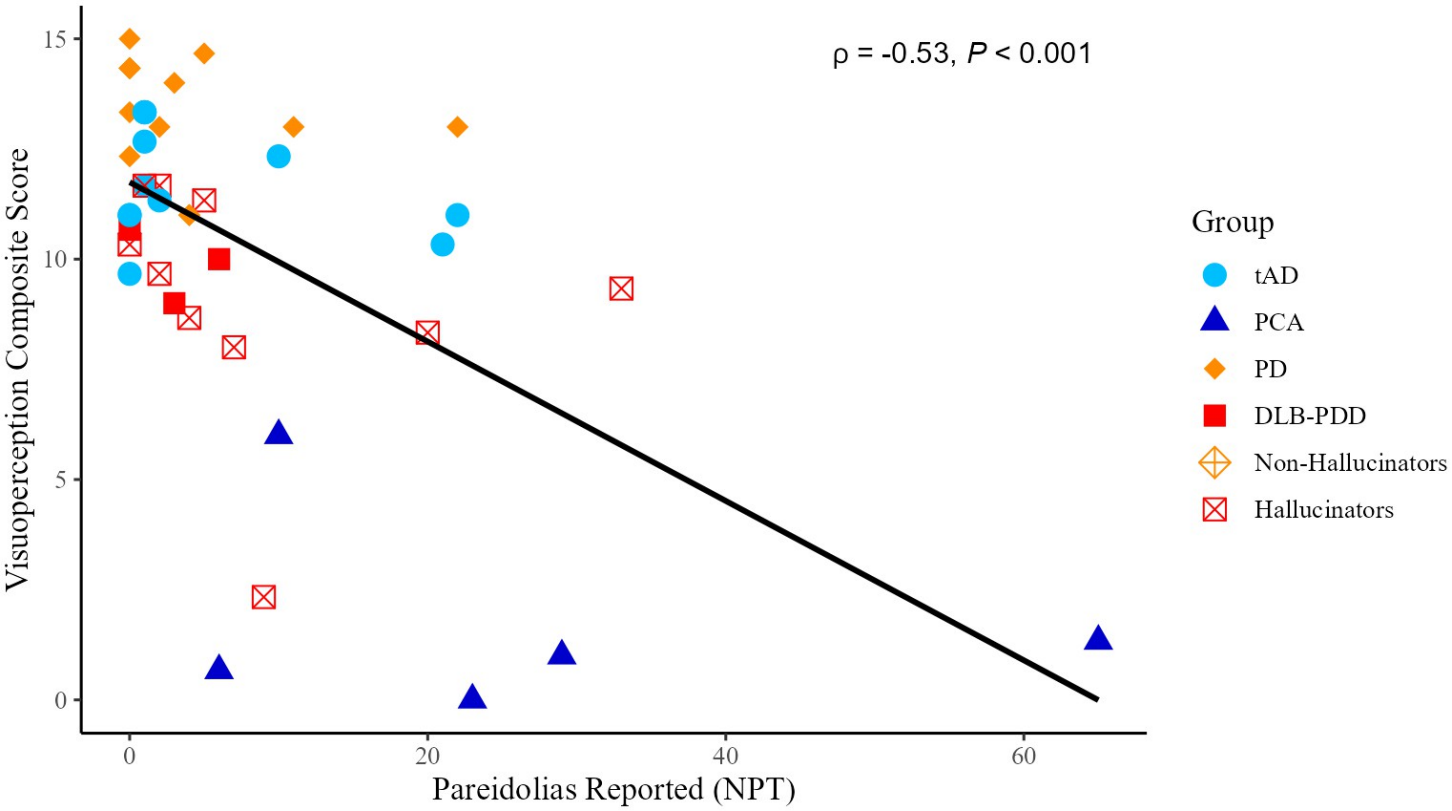
Spearman’s correlation between visuoperception and pareidolic responses.

Spearman’s correlation showed a significant relationship between the composite visuoperception score and pareidolic responses (ρ = - 0.53, *p* < 0.001; Fig 3). Pareidolic responses also significantly correlated with each of the individual components of the composite score: the Incomplete Letters (ρ = - 0.53, *p* = 0.001); Cube Analysis (ρ = - 0.42, *p* = 0.005); and Progressive Silhouettes VOSP subtests (ρ = - 0.50, *p* < 0.001).

### Regression in all patient groups

The linear regression model, run to test whether VH or visuoperception predict the number of pareidolic responses, explained a significant amount of variance in pareidolic responses, F(3, 39) = 6.86, *p* < 0.001, R^2^ = 0.30. The composite visuoperception score was a significant predictor of pareidolic responses (β = - 0.62, *t*(36) = - 4.48, *p* < 0.001) whereas history of VH was not (β = 0.35, *t*(36) = 0.80, *p* = 0.35). A decrease of one visuoperception point corresponded to an average increase in pareidolic responses by 1.98 with 95% confidence [- 2.87, -1.08]. Re-running these regressions with age and sex as co-variates of no interest had no impact on the results.

## Discussion

We investigated whether pareidolic responses on the NPT were associated with VH presence, or visuoperceptual impairment in LBDs. In contrast to previous findings, there was no relation between pareidolic responses and history of VH in patients with an LBD. LBD patients with a history of VH had worse global cognitive scores and visuoperceptual impairments compared to those without VH. Visuoperceptual impairment, not VH, predicted pareidolic responses across all patient groups. Increased pareidolic responses were particularly prevalent in the PCA group which was characterized by extreme visuoperceptual dysfunction without VH. Overall, the analyses consistently pointed to pareidolias being the result of visuoperceptual impairment but not directly associated with VH.

VH are a strong diagnostic predictor for the development of DLB and PDD [2, 32, 33]. Yokoi et al. [9] proposed that pareidolias are complex visual illusions that share a common underlying neural and psychological mechanism with VH. The NPT was designed to evoke this misperception in LBD patients with pareidolias being a surrogate marker for VH. Yokoi et al. (2014) had three major findings: DLB patients had more pareidolic responses compared to tAD patients; pareidolic responses in DLB correlated with informant-corroborated accounts of VH; and, both pareidolic responses and VH improved after DLB patients started the cholinesterase inhibitor, donepezil. They also reported that pareidolic responses in DLB correlated with multiple measures of visuoperception, and this correlation was greater for those not taking donepezil. While Yokoi et al. [9] concluded that these findings indicated a common psychological and neural mechanism for VH and pareidolias, the current findings strongly suggest an alternate explanation—that being, that pareidolias arise as a consequence of visuoperceptual impairment; their association, in turn, with VH being a consequence of VH also being associated with visuoperceptual impairment. This interpretation finds considerable resonance with recent studies. One reported that, while pareidolias were common in cognitively impaired Lewy body patients, this was equally the case in the subgroup with no history of hallucination [34]. Another found that hallucinating PD patients reported more pareidolic responses but also showed greater impairments on visuoperception tests compared to PD without hallucination [35].

It has also been reported that pareidolic responses could discriminate between DLB and tAD patients with the latter not hallucinating and reporting significantly less pareidolias when compared to DLB [9, 13]. In Mild Cognitive Impairment (MCI) stages, however, pareidolic responses had little sensitivity for separating DLB from tAD [13]. The tAD patients in the present study also reported less pareidolias compared to the DLB-PDD group—though this did not reach significance—partially replicating the results of Yokoi et al. [9]. In both studies, the tAD group showed only mild visuoperceptual deficits. In contrast, in the present study, PCA with AD pathology demonstrated a complete dissociation between pareidolias and VH; PCA reported the most pareidolias of all patient groups, without reporting any VH. This observation offers strong evidence that pareidolic responses are a consequence of impaired visuoperception.

Seemingly at odds with the current interpretation, Yokoi et al. [9] reported that DLB patients who started a cholinesterase inhibitor saw an improvement in both VH and pareidolias, but not on tests of visuoperception. This is surprising given that cholinesterase inhibitors are known to improve functionality of brain regions involved in visuoperception [6, 36, 37]. Performance on the visuoperceptual tests used by Yokoi et al., however, was close to ceiling whereas the pareidolia scores were well above floor. Therefore, a plausible explanation for their results might be that there was room to improve pareidolias (due to absence of floor effects) but not so visuoperceptual scores (due to ceiling effects) with cholinesterase inhibitors in their study.

A limitation of the present study was the sample size for each disease group. This study, however, included two pathologies and four different syndromic groups; we were able to contrast performance between LBD (PD, PDD and DLB) and Alzheimer pathology (tAD and PCA) to identify the associations of pareidolias with very robust statistical effects. The finding that LBD with VH had worse visuoperceptual performance than those without VH was an obvious result when one considers that 77% of the DLB-PDD group had a history of VH whereas none of the PD group had a history of VH in the present study. In other words, the VH positive LBD group was mostly comprised of patients with dementia while the VH negative LBD group was mostly PD patients without dementia, making this result unavoidable and thus a study limitation. The importance of this particular finding, however, was not the obvious and rather circular observation that LBD patients with dementia have worse visuoperceptual ability than those without dementia. Rather it was that despite this being the case, there was no significant difference in the pareidolia score between LBD groups stratified for VH status, thus offering strong evidence that pareidolic scores could not be a surrogate marker for VH.

The classification of VH status in patients taking a cholinesterase inhibitor could potentially have been another limitation. Cholinesterase inhibitors are prescribed for cognitive impairment and also suppress VH in LBD [38]. Thus, it is conceivable that classification of participants as VH negative could be confounded by chronic cholinesterase inhibitor use. It is highly unlikely that this would have influenced the results in the current study however, because there was only one LBD patient who had been chronically taking a cholinesterase inhibitor with no history of VH. Removing this patient from the analyses had no impact on the findings of the study. It is also unclear whether other methods of pareidolia elicitation [10, 11] might yield differing results from those found in this study with the NPT; importantly, our results highlight that future studies using other techniques need to carefully ensure that the confound of visuoperceptual dysfunction is accounted for before attributing test performance to a VH tendency.

While pareidolias and VH were proposed to be similar phenomena with a common underlying mechanism [8, 9], the current study found that these phenomena can be dissociated from each other. Furthermore, the current findings strongly argue that pareidolias are primarily a consequence of impaired visuoperceptual ability. These results should not be interpreted, however, as suggesting that measuring pareidolias with the NPT is invalid—it was sensitive to DLB-PDD as has been reported previously [7, 9, 34]. It should not, however, be considered a surrogate for VH.
